# Platelet Mass Signatures and Thrombin-Activatable Fibrinolysis Inhibitor as Discriminators of Primary Thrombocytosis: Development of a Screening Algorithm in an Eastern Sudanese Cohort

**DOI:** 10.7759/cureus.106174

**Published:** 2026-03-31

**Authors:** Bashir A Bashir

**Affiliations:** 1 Department of Hematology, Faculty of Medical Laboratory Sciences, Port Sudan Ahlia University, Port Sudan, SDN

**Keywords:** diagnostic model, plateletcrit, platelet indices, roc curve, tafi, thrombocytosis

## Abstract

Background: Clinically differentiating primary from reactive thrombocytosis is crucial, but it often requires expensive molecular and bone marrow examinations. Platelet indices and thrombosis-associated biomarkers may offer valuable diagnostic assistance.

Objective: This study aims to evaluate platelet indices and thrombin-activatable fibrinolysis inhibitor (TAFI) for discrimination between primary and reactive thrombocytosis and to develop a combined diagnostic model.

Methods: A cross-sectional analytical study included 74 Sudanese patients with thrombocytosis (29 primary and 45 reactive). Platelet indices and TAFI were measured. Diagnostic performance was assessed using the receiver operating characteristic (ROC) curve analysis and multivariable logistic regression.

Results: Primary thrombocytosis exhibited markedly elevated platelet count (PLT), plateletcrit (PCT), platelet large cell count (PLCC), and TAFI levels (all p < 0.001). ROC analysis indicated the highest discrimination for TAFI (area under the curve (AUC): 0.925), plateletcrit (AUC: 0.916), and platelet count (AUC: 0.890). The optimal TAFI threshold of 24.6 resulted in a sensitivity of 82.8% and a specificity of 97.8%. A composite multi-marker model (PLT + PCT + PLCC + TAFI) attained an AUC of 0.975.

Conclusion: Plateletcrit and TAFI are powerful discriminators of primary thrombocytosis. A combined marker model offers superior diagnostic accuracy and may function as a cost-effective screening technique in settings with limited resources.

## Introduction

Thrombocytosis is characterized by a platelet count beyond 450 × 10⁹/L and is generally categorized into primary (clonal) or reactive (secondary) [1]. Primary thrombocytosis predominantly arises in the context of myeloproliferative neoplasms, especially essential thrombocythemia, and results from autonomous megakaryocytic proliferation driven by acquired genetic alterations [2]. Reactive thrombocytosis occurs when thrombopoiesis is secondarily activated by infection, inflammation, iron deficiency, cancer, or tissue injury, usually via cytokine signaling pathways [3,4].

Accurate distinction between clonal and reactive thrombocytosis is clinically significant because of the substantial differences in risk profiles and therapeutic approaches. Primary thrombocytosis is linked to heightened thrombotic and hemorrhagic risks. It may necessitate cytoreductive or targeted therapy, whereas reactive thrombocytosis is often managed by treating the underlying illness and rarely requires platelet-directed therapy [2,5].

Present diagnostic validation of clonal thrombocytosis depends on bone marrow analysis and molecular assessment for driver mutations, including Janus kinase 2 (JAK2), calreticulin (CALR), and myeloproliferative leukemia (MPL) [2,6]. While diagnostically conclusive, these studies are expensive and reliant on infrastructure, and their availability varies significantly across regions. This presents significant diagnostic hurdles and may delay appropriate risk categorization and treatment decisions [7]. Consequently, there is growing interest in scalable, laboratory-based biomarkers that can facilitate early diagnostic stratification using readily available technologies.

Nowadays, automated hematology analyzers produce extended platelet parameters, such as mean platelet volume (MPV), platelet distribution width (PDW), plateletcrit (PCT), platelet large cell count (PLCC), and platelet large cell ratio (PLCR), that indicate platelet size variability and overall platelet mass [8]. These indices are cost-effective and readily accessible, and require no additional laboratory resources beyond conventional whole-blood count testing. Numerous studies have indicated that platelet biomass and size distribution patterns vary between clonal and reactive thrombopoiesis, suggesting potential diagnostic significance [8,9].

Concurrently, thrombin-activatable fibrinolysis inhibitor (TAFI) has emerged as a significant modulator of the fibrinolytic equilibrium and thrombotic propensity, with potential implications for clonal platelet diseases and prothrombotic conditions [9]. Integrating platelet indices with fibrinolysis-related indicators may enhance discriminatory performance while ensuring feasibility in standard laboratory operations.

Research assessing platelet indices as diagnostic differentiators in African populations is still scarce. Producing locally sourced diagnostic performance data and algorithm-driven screening methods may enhance practical, cost-efficient diagnostic routes and facilitate wider access to appropriate hematologic assessments.

Prior research has examined the use of platelet indices to distinguish clonal from reactive thrombocytosis. Many studies show that measures such as plateletcrit, mean platelet volume, and platelet distribution width serve as effective individual markers [10-12]. More recent work has aimed to improve diagnosis by combining platelet indices with clinical factors in scoring systems and models [12,13]. While these methods perform well, they mostly rely on platelet indices and do not include markers that show other disease processes. Studies focused on fibrinolysis indicators, such as thrombin-activatable fibrinolysis inhibitor (TAFI), are rare. Thus, adding platelet mass indices to fibrinolytic markers may provide additional information and help better differentiate between primary and reactive thrombocytosis.

Accordingly, this study aimed to evaluate the diagnostic performance of platelet indices and TAFI in differentiating primary from reactive thrombocytosis and to develop a combined diagnostic model intended as a screening and triage tool for prioritizing patients for confirmatory evaluation in an eastern Sudanese cohort.

## Materials and methods

Study design and setting

This cross-sectional analytical investigation involved individuals with documented thrombocytosis who were assessed at the Hematology Department of Port Sudan Ahlia University. Patients were categorized into main and reactive thrombocytosis groups according to clinical and hematologic evaluation. Data were collected from July 2024 to December 2025, and only cases with comprehensive laboratory and diagnostic categorization data were included in the final analysis.

Study population

A total of 74 patients with confirmed thrombocytosis were included in the analysis. Thrombocytosis is defined as a platelet count exceeding 450 × 10⁹/L, as measured by automated hematology analysis.

Patients were classified into two diagnostic groups. The primary thrombocytosis cohort was diagnosed based on clinical observations, laboratory findings, and expert hematologic evaluation indicative of a clonal myeloproliferative disorder. Molecular testing (e.g., JAK2, CALR, and MPL) and/or bone marrow examination were performed when clinically indicated and available but were not consistently used in all cases.

The reactive thrombocytosis group included patients with secondary thrombocytosis when thrombocytosis occurred in association with an identifiable secondary cause, such as infection, inflammation, iron deficiency, tissue injury, or malignancy, and when clonal disease was clinically excluded. Only cases with complete platelet index, TAFI measurements, and confirmed diagnostic classification were retained.

Laboratory measurements

Complete blood counts (CBCs) and platelet indices were assessed using an automated hematology analyzer (DH 800, Dymind, Shenzhen, China), following the manufacturer's instructions. The analyzers measure and report platelet count, mean platelet volume (MPV), platelet distribution width (PDW), plateletcrit (PCT), platelet large cell count (PLCC), and platelet large cell ratio (PLCR). These values are measured using automated impedance or optical methods. Routine internal quality control was performed throughout the study period.

Plasma thrombin-activatable fibrinolysis inhibitor (TAFI) levels were quantified using a commercial enzyme-linked immunosorbent assay (ELISA) kit (Human CPB2/TAFI ELISA, Cloud-Clone Corp, Wuhan, China). The manufacturer's guidelines were followed. Samples and standards were analyzed in duplicate. Absorbance was measured at 450 nm with a microplate reader. Concentrations were determined using a standard calibration curve. All assays were conducted in a regulated laboratory setting, in compliance with the kit's quality control guidelines.

Statistical analysis

Statistical analysis was conducted using the standard Statistical Package for the Social Sciences (SPSS) software version 25 (IBM Corp., Armonk, NY). The distribution of continuous variables was evaluated using the Kolmogorov-Smirnov and Shapiro-Wilk tests. Due to significant deviation from normality across all major variables (p < 0.05), non-parametric statistical methods were consistently used. Continuous variables are shown as median and interquartile range (IQR). Categorical variables are represented as frequencies and percentages. Comparisons between primary and reactive thrombocytosis groups were conducted using the Mann-Whitney U test for continuous variables. A two-tailed p-value of less than 0.05 was deemed statistically significant.

Associations between platelet count and platelet indices were assessed using Spearman's rank correlation coefficients because of the non-normal distribution of the data. The strength of correlation was assessed using established standards for non-parametric coefficients. The diagnostic efficacy of each laboratory parameter for diagnosing primary thrombocytosis was assessed by receiver operating characteristic (ROC) curve analysis. ROC curves were generated for each marker (PLT, PCT, PLCC, MPV, PDW, PLCR, age, and TAFI).

For each ROC curve, the following metrics were computed: the area under the curve (AUC), the optimal cutoff determined by the Youden index, and sensitivity and specificity at this cutoff. Each marker was evaluated separately to ascertain its individual diagnostic efficacy. A multivariable logistic regression model was developed to assess the combined diagnostic performance of selected high-performing indicators (PLT, PCT, PLCC, and TAFI) while adjusting for age. Multicollinearity was evaluated using correlation analysis, and variables were selected based on diagnostic performance and biological relevance. Probabilities from the model were utilized to produce a composite ROC curve and the associated AUC.

Model discrimination was evaluated by ROC analysis of projected probabilities. The integrated model was evaluated against the performance of separate markers. A diagnostic screening strategy was constructed utilizing ROC-derived threshold values for platelet count, plateletcrit, platelet large cell count, and TAFI. Thresholds were determined based on optimal combined sensitivity and specificity and arranged into a sequential clinical decision flowchart designed for laboratory screening applications. All statistical tests were two-tailed, with statistical significance established at p < 0.05.

Ethical approval

Ethical approval was obtained from the Research Ethics Committee (REC) of Port Sudan Ahlia University (PAU), Port Sudan, Sudan (approval number: REC-PAU 3/5). The study was conducted in accordance with the Declaration of Helsinki. Written informed consent was obtained from all participants or their legal guardians prior to enrollment and sample collection.

## Results

A total of 74 patients with thrombocytosis were included, consisting of 29 (39.2%) with primary thrombocytosis and 45 (60.8%) with reactive thrombocytosis. The sex distribution did not differ significantly between the primary and reactive thrombocytosis groups. In the primary thrombocytosis cohort, 14 of 29 (48.3%) were male, and 15 of 29 (51.7%) were female; in the reactive thrombocytosis group, 18 of 45 (40%) were male, and 27 of 45 (60%) were female. The sex distribution did not differ significantly between the primary and reactive thrombocytosis groups (χ²(1) = 0.213, p = 0.645, Cramér's V = 0.054), indicating a negligible association.

Table 1 indicates that patients with primary thrombocytosis were substantially older than those with reactive thrombocytosis, with a median age of 42 years compared to 14 years (p = 0.003). The platelet count was significantly elevated in the main group (median: 889 × 10⁹/L) relative to the reactive group (median: 551 × 10⁹/L, p < 0.001). Platelet mass indices exhibited substantial differences: plateletcrit (PCT) was elevated in primary thrombocytosis (median: 0.735 versus 0.452, p < 0.001), and platelet large cell count (PLCC) was similarly greater in primary thrombocytosis (median: 135 versus 73, p < 0.001). Conversely, MPV and PDW exhibited no significant differences across the groups (p = 0.119 and p = 0.310, respectively). PLCR exhibited a non-significant tendency toward elevated levels in primary thrombocytosis (p = 0.077). TAFI levels revealed a significant difference between the primary and reactive thrombocytosis groups (p < 0.001). Descriptive analysis revealed significantly elevated TAFI values in primary thrombocytosis, with a median of 29.4 (IQR: 24.7-38.2), compared with 13.8 (IQR: 9.0-19.4) in reactive thrombocytosis.

**Table 1:** Comparison of platelet indices and TAFI levels in primary and reactive thrombocytosis The data has been represented as number, %, median, and IQR; the p-value is considered significant at <0.05. PLT: platelet count, MPV: mean platelet volume, PDW: platelet distribution width, PCT: plateletcrit, PLCC: platelet large cell count, PLCR: platelet large cell ratio, TAFI: thrombin-activatable fibrinolysis inhibitor, IQR: interquartile range

Parameter	Primary thrombocytosis (n = 29), median (IQR)	Reactive thrombocytosis (n=45), median (IQR)	p-value
Age (years)	42 (16-52)	14 (2-33)	0.003
Sex			0.645
Male	14 (48.3%)	18 (40%)	
Female	15 (51.7%)	27 (60%)	
PLT (×10⁹/L)	889 (747-1031)	551 (515-622)	<0.001
MPV (fL)	8.3 (7.8-9.0)	7.9 (7.5-8.5)	0.119
PDW (fL)	15.6 (15.0-16.3)	15.4 (15.1-15.6)	0.31
PCT (%)	0.735 (0.620-0.880)	0.452 (0.410-0.526)	<0.001
PLCC (×10⁹/L)	135 (112-185)	73 (61-97)	<0.001
PLCR (%)	17.2 (13.8-22.6)	13.0 (11.3-17.7)	0.077
TAFI (ng/mL)	29.4 (24.7-38.2)	13.8 (9.0-19.4)	<0.001

Spearman's correlation analysis revealed a very strong link between platelet count and plateletcrit (ρ = 0.907, p < 0.001) and a substantial correlation with PLCC (ρ = 0.614, p < 0.001). In contrast, the correlations between platelet count and MPV, PDW, PLCR, and age were not significant (p > 0.05).

A multivariable logistic regression model was developed with primary thrombocytosis as the dependent variable. Platelet count, plateletcrit, platelet large cell count, mean platelet volume (MPV), platelet distribution width (PDW), platelet large cell ratio (PLCR), and age were included as predictors. The model exhibited robust discriminative performance and was statistically significant (likelihood ratio test, p < 0.001), with a pseudo-R² of roughly 0.75, indicating considerable explanatory power.

Following multivariable adjustment, platelet count, plateletcrit, MPV, and age were independently correlated with primary thrombocytosis. The PLT had a notable positive correlation with clonal thrombocytosis (p = 0.011). Plateletcrit remained an independent discriminator (p = 0.037). MPV had an independent effect after adjustment (p = 0.043). This finding indicates that age was accounted for in the multivariable model and did not fully explain the observed associations between platelet indices, TAFI, and thrombocytosis type. Conversely, PLCC, PDW, and PLCR lost independent significance in the multivariable model following adjustment for correlated platelet mass characteristics.

ROC curve analysis was conducted to assess diagnostic discrimination for important indices, with diagnostic performance parameters provided in Table 2. Plateletcrit had the highest accuracy (AUC: 0.916), followed by platelet count (AUC: 0.890) and PLCC (AUC: 0.856). Employing the Youden index to determine appropriate thresholds, a PCT cutoff of 0.59 attained 86.2% sensitivity and 93.3% specificity, and a platelet count cutoff of 713 × 10⁹/L produced 86.2% sensitivity and 88.9% specificity. PLCC at a cutoff of 100 yielded 86.2% sensitivity and 75.6% specificity. Age exhibited relatively moderate discriminating ability (AUC: 0.704).

**Table 2:** ROC curve analysis of laboratory markers for discriminating primary from reactive thrombocytosis p-value is considered significant at <0.05. PLT: platelet count, MPV: mean platelet volume, PDW: platelet distribution width, PCT: plateletcrit, PLCC: platelet large cell count, PLCR: platelet large cell ratio, TAFI: thrombin-activatable fibrinolysis inhibitor, AUC: area under the curve, CI: confidence interval, ROC: receiver operating characteristic

Marker	AUC	p-value	95% CI	Best cutoff	Sensitivity	Specificity
TAFI	0.925	0.000	0.860-0.990	24.6	82.80%	97.80%
PCT	0.916	0.000	0.843-0.989	0.59	86.20%	93.30%
PLT	0.89	0.000	0.796-0.986	713	86.20%	88.90%
PLCC	0.856	0.000	0.772-0.940	100	86.20%	75.60%
Age	0.704	0.003	0.575-0.834	37	65.50%	82.20%
PLCR	0.622	0.077	0.485-0.759	13.8	75.90%	57.80%
MPV	0.608	0.120	0.473-0.742	8.1	65.50%	57.80%
PDW	0.57	0.311	0.421-0.719	16.1	41.40%	91.10%

**Figure 1:**
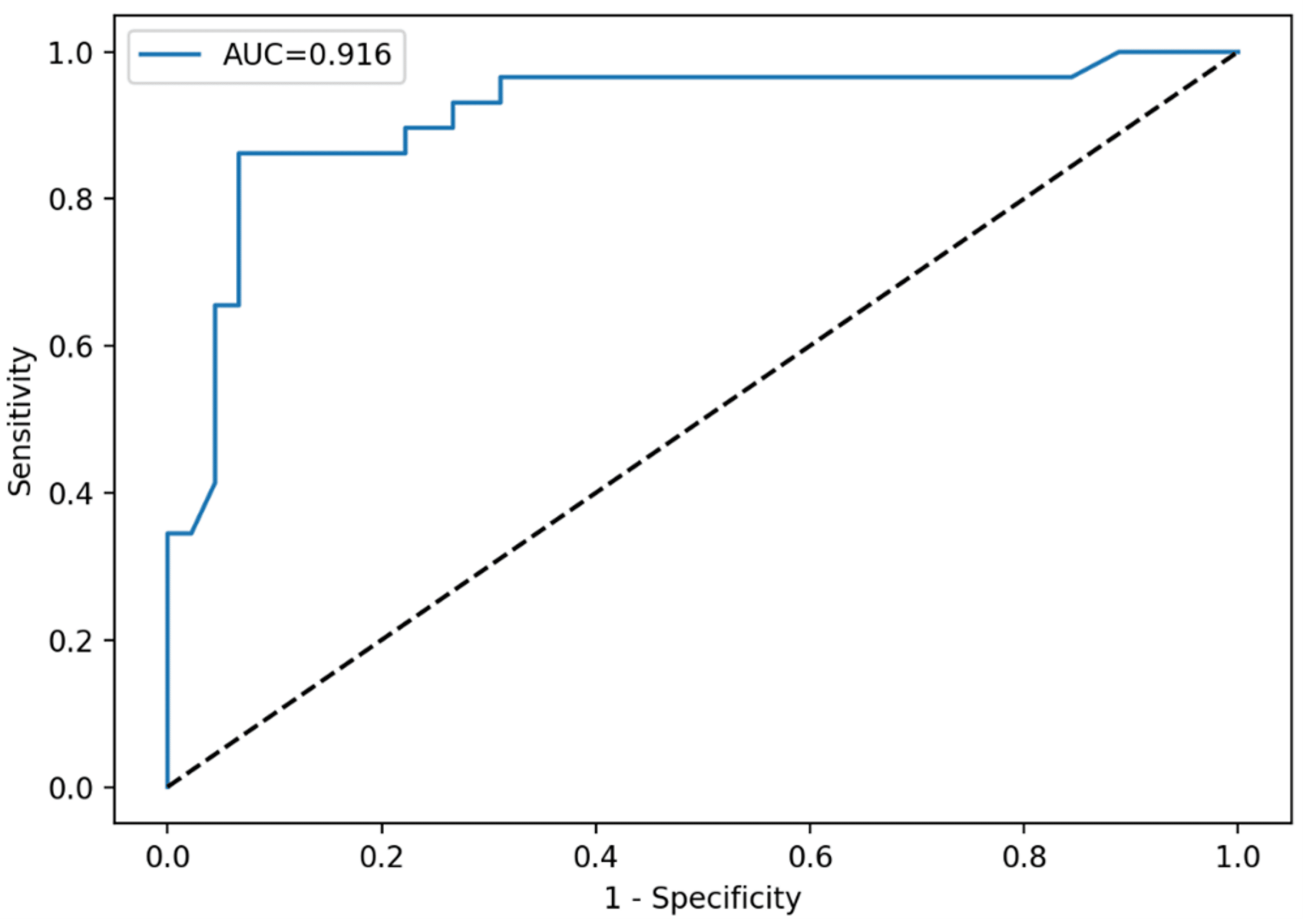
ROC curve for PCT demonstrated excellent diagnostic performance with an AUC of 0.916 ROC: receiver operating characteristic, PCT: plateletcrit, AUC: area under the curve

**Figure 2:**
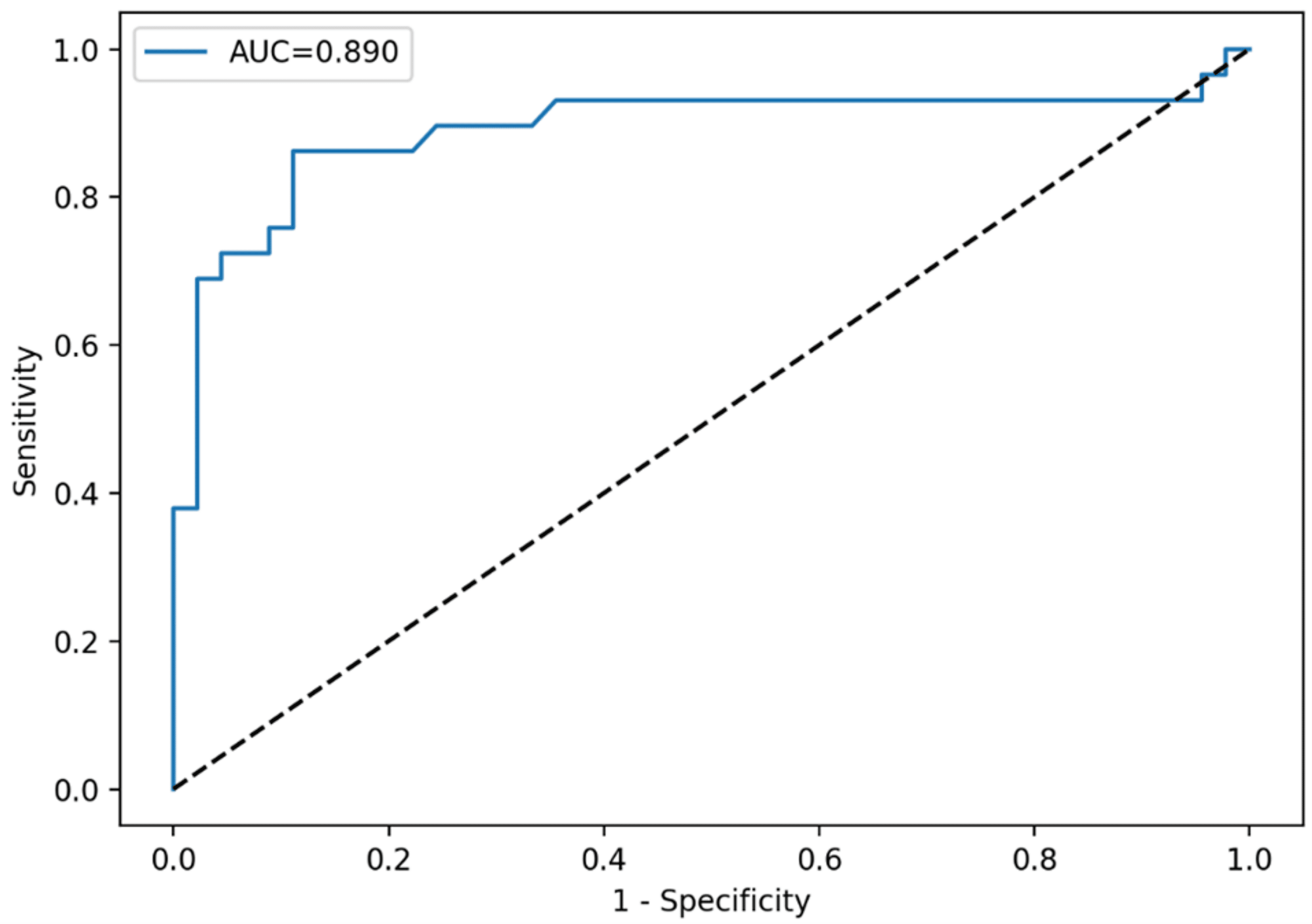
ROC curve for PLT demonstrated good diagnostic performance with an AUC of 0.890 ROC: receiver operating characteristic, PLT: platelet count, AUC: area under the curve

**Figure 3:**
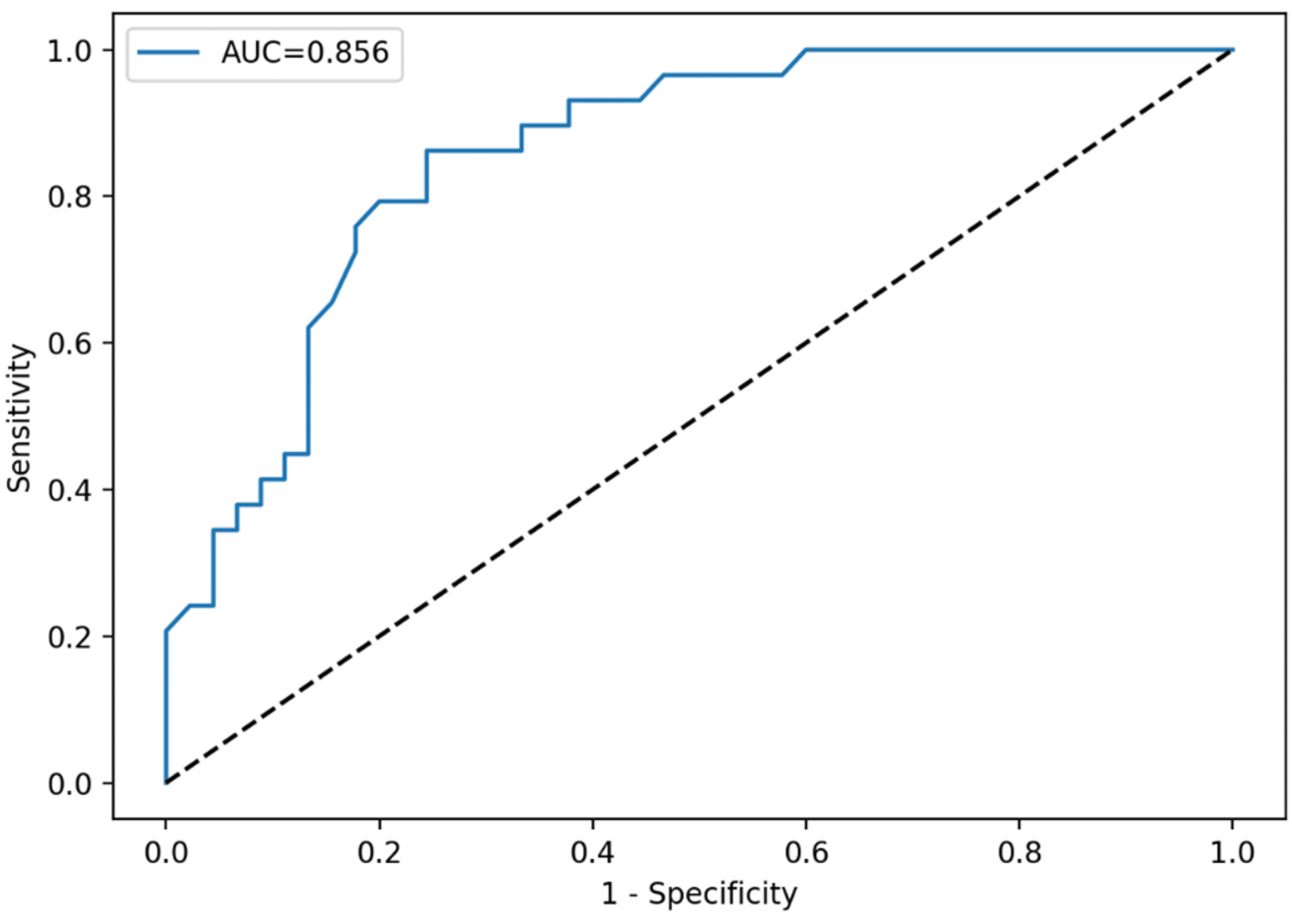
ROC curve of PLCC in thrombocytosis classification: PLCC demonstrated good diagnostic performance with an AUC of 0.856 ROC: receiver operating characteristic, PLCC: platelet large cell count, AUC: area under the curve

**Figure 4:**
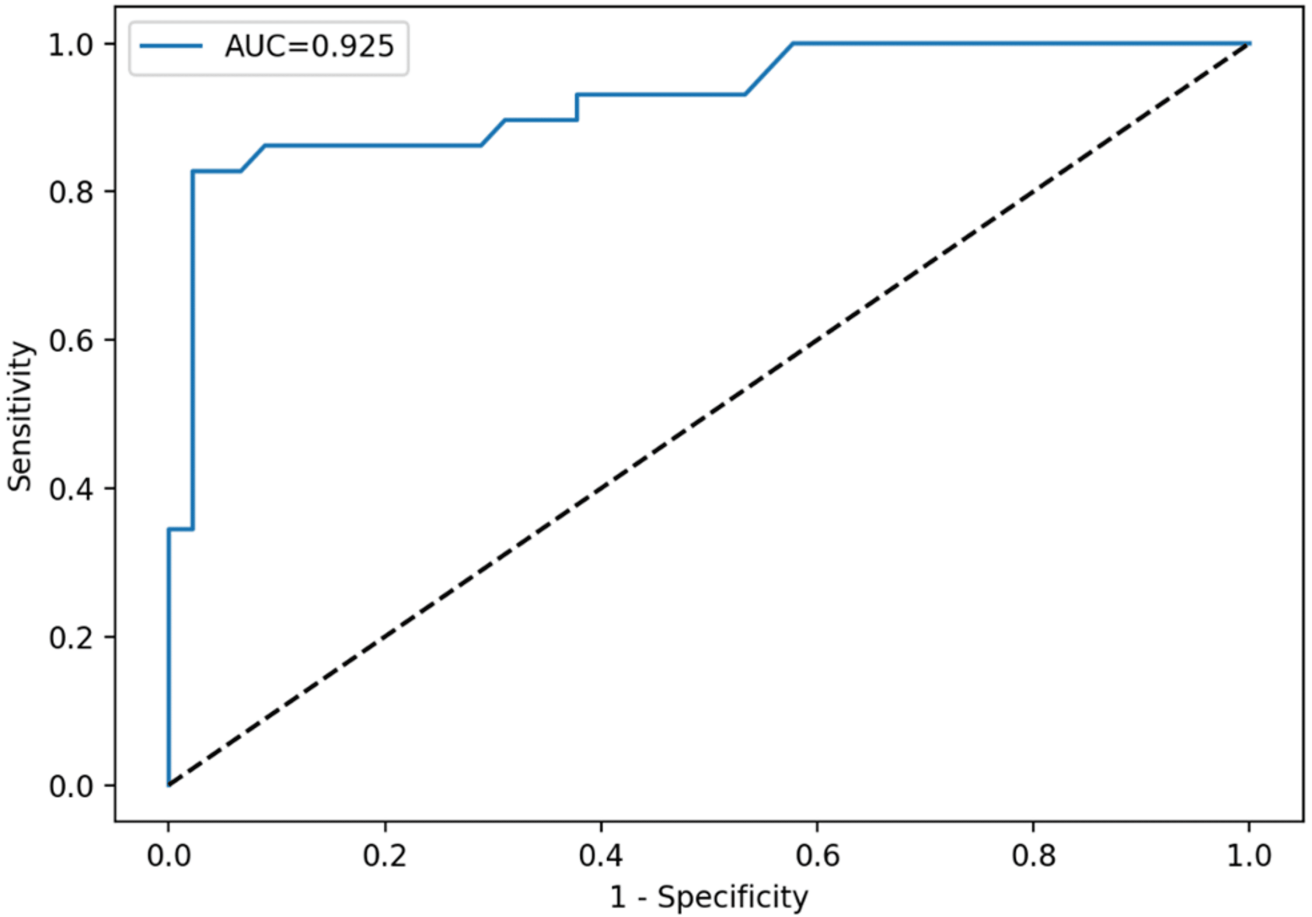
Diagnostic ROC curve of TAFI in differentiating primary and reactive thrombocytosis: TAFI demonstrated excellent diagnostic performance with an AUC of 0.925 ROC: receiver operating characteristic, TAFI: thrombin-activatable fibrinolysis inhibitor, AUC: area under the curve

ROC curve analysis demonstrated that TAFI provided the highest diagnostic accuracy for discriminating primary from reactive thrombocytosis, with an AUC of 0.925. A cutoff value of 24.6 yielded 82.8% sensitivity and 97.8% specificity. MPV, PDW, and PLCR demonstrated limited discriminatory ability (AUC: <0.65). Individual ROC curves for each parameter are presented in Figures 1-4.

**Figure 5:**
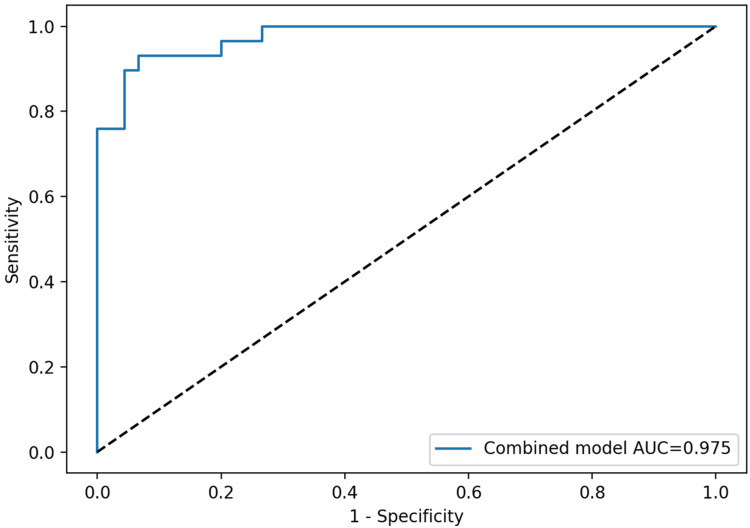
ROC curve of the platelet mass signature and TAFI combined diagnostic model, combining platelet count, plateletcrit, platelet large cell count, and TAFI: the integrated model demonstrates excellent discrimination between primary and reactive thrombocytosis (AUC: 0.975) ROC: receiver operating characteristic, TAFI: thrombin-activatable fibrinolysis inhibitor, AUC: area under the curve

Predicted probabilities obtained from the multivariable model integrating platelet count, plateletcrit, platelet large cell count, and TAFI were further employed to generate a composite ROC curve. This multi-marker model demonstrated outstanding diagnostic efficacy, with an AUC of 0.975, surpassing that of any individual marker (Figure 5).

A progressive laboratory screening algorithm was developed using ROC-derived cutoff values and multivariable model performance to facilitate the practical separation between primary and reactive thrombocytosis. The algorithm incorporates platelet count, plateletcrit, platelet large cell count, and TAFI thresholds determined from diagnostic performance analysis. The sequential application of these markers enhances classification accuracy beyond that of any individual characteristic.

A stepwise laboratory screening approach was developed based on ROC-derived thresholds and multivariable model performance. Initially, thrombocytosis is confirmed by a platelet count exceeding 450 × 10⁹/L. Platelet mass indices are then evaluated, where elevated plateletcrit (≥0.59), platelet count (≥713 × 10⁹/L), and platelet large cell count (≥100 × 10⁹/L) suggest a higher likelihood of primary thrombocytosis. In such cases, TAFI measurement is performed; levels ≥24.6 ng/mL strongly support a clonal process. Finally, a combined interpretation of these markers improves diagnostic confidence and identifies patients who may benefit from confirmatory molecular or bone marrow evaluation.

## Discussion

This study reveals that, in a Sudanese population, indices linked to platelet mass and TAFI differ significantly between primary and reactive thrombocytosis, and that a combined model integrating these markers provides superior diagnostic differentiation. Plateletcrit and PLCC consistently distinguished the two groups, although size variability indicators (MPV, PDW, and PLCR) were less reliable in this context.

In recent years, platelet indices such as plateletcrit, PDW, and MPV have been increasingly studied as cost-effective, readily accessible biomarkers for differentiating the origins of quantitative platelet abnormalities. Automated analyzers deliver these indices within standard complete blood count panels, and numerous studies have highlighted their effectiveness in distinguishing clonal from reactive situations in various contexts [10,11]. A recent cross-sectional investigation demonstrated significantly elevated plateletcrit and platelet distribution width in clonal thrombocytosis compared to reactive thrombocytosis, supporting our findings that platelet biomass measurements are superior to size metrics alone [12].

The observed elevation of plateletcrit in primary thrombocytosis is scientifically justifiable: clonal megakaryopoiesis results in an augmented reservoir of circulating platelets, leading to an enhanced total platelet mass. This corresponds with previous research demonstrating the diagnostic significance of plateletcrit in myeloproliferative disorders, with suggested cutoff criteria that partially coincide with those established in our ROC analysis [12]. Although MPV and PDW may vary across thrombocytosis subtypes, their efficacy is inconsistent and often affected by the analyzer platform and clinical context [10,13], thereby elucidating their restricted discriminative ability in this population.

TAFI proved to be a highly effective discriminator in our sample, with an AUC of 0.925 on the ROC curve. TAFI mechanistically modulates fibrinolysis by influencing fibrin stability, and elevated TAFI activity has been linked to prothrombotic phenotypes in several clinical populations [14]. The robust performance shown indicates that fibrinolytic balance markers may enhance platelet indices in elucidating the underlying pathobiology of clonal thrombocytosis, beyond mere platelet volume or count.

The strong diagnostic efficacy of TAFI aligns with prior findings, which specifically show increased TAFI activity and markedly elevated TAFI levels in clonal thrombocytosis compared to reactive thrombocytosis. This distinction supports TAFI's involvement in the prothrombotic phenotype of myeloproliferative diseases. TAFI is a crucial inhibitor of fibrinolysis: it stabilizes fibrin clots and diminishes plasmin-mediated breakdown [14]. In clonal thrombocytosis, heightened thrombin generation modifies the hemostatic equilibrium and can augment TAFI activation, leading to a hypofibrinolytic and prothrombotic state. The pathophysiological differences between clonal and reactive thrombocytosis may help explain the pronounced selective capacity of TAFI found in our investigation.

The multivariable model that combined platelet count, plateletcrit, PLCC, and TAFI yielded the greatest discrimination (AUC: 0.975), highlighting the significance of integrated, multi-marker methodologies. This illustrates a broader trend in hematologic diagnostics: although individual indices often yield insufficient information, integrating biomarkers that represent distinct biological processes can significantly enhance classification accuracy [15]. Integrated models are especially beneficial in contexts where molecular and marrow analyses are costly or logistically challenging to obtain.

The algorithm based on ROC-optimized thresholds provides a pragmatic clinical screening framework. By employing regular analyzer results enhanced by TAFI, doctors can more effectively stratify patients before doing more invasive or resource-demanding confirmation procedures. This technique is consistent with recent research advocating the sequential application of laboratory indicators to inform decision-making in thrombocytosis and associated hematologic diseases [10,12].

The suggested screening algorithm can be seamlessly integrated into standard laboratory operations with negligible added burden. The initial assessment commences with a standard complete blood count (CBC). Platelet indices, namely, platelet count, plateletcrit, and platelet large cell count, are automatically produced by contemporary hematology analyzers. Cases surpassing ROC-derived platelet mass thresholds may then undergo targeted TAFI testing as a secondary laboratory procedure. Patients who meet the combined marker requirements may be prioritized for confirmatory molecular testing and/or bone marrow evaluation. This sequential methodology (CBC → platelet indices → TAFI → specialist referral) is intended for initial screening and risk stratification rather than definitive diagnosis. It facilitates prompt diagnostic stratification while maximizing the utilization of advanced diagnostic tools.

Ultimately, establishing population-specific thresholds shows the significance of local validation. The majority of current research on platelet indices and thrombocytosis has focused on European or Asian cohorts, with limited investigations of these associations in African populations. Establishing locally pertinent thresholds improves both clinical relevance and fairness in diagnostic processes.

Several limitations should be acknowledged. The sample size was moderate and derived from a single regional cohort, potentially limiting generalizability. The integrated diagnostic model demonstrated outstanding discriminative efficacy; however, internal validation techniques such as cross-validation or bootstrapping were not used. Consequently, the reported AUC may be subject to optimism bias and could overestimate performance in external populations. The marked age difference between groups may confound the analysis. Although age was included in the multivariable model, age-adjusted ROC analyses were not separately performed, and residual confounding cannot be entirely excluded. Larger, multicenter studies are necessary for broader validation. Additionally, platelet indices depend on the analyzer used, and variability among instruments may affect comparisons across different sites and platforms. Furthermore, the model does not account for molecular mutation status (e.g., JAK2, CALR, or MPL) nor does it include bone marrow morphology. Additionally, confirmatory diagnostic techniques, including molecular testing and bone marrow examination, were not consistently conducted among all patients. This variability may lead to bias in categorization between the main and reactive thrombocytosis categories. Therefore, its effectiveness within molecularly defined disease categories remains undetermined. Although TAFI demonstrated strong discriminatory ability, concerns about assay variability and consistency require further investigation before widespread clinical adoption. Formal multicollinearity diagnostics, such as the variance inflation factor, were not performed, and residual collinearity among platelet indices cannot be ruled out entirely. The findings should therefore be interpreted with caution, and external validation in independent cohorts is required before broader clinical implementation.

## Conclusions

In this cohort, plateletcrit, platelet count, PLCC, and particularly TAFI accurately differentiated primary from reactive thrombocytosis. TAFI exhibited the most robust individual diagnostic efficacy, while a combined model incorporating platelet mass and fibrinolysis indicators attained exceptional accuracy. While these findings support a practical and cost-effective approach using standard analyzer indices and TAFI to prioritize patients for molecular and marrow assessment, external validation in larger, independent cohorts is required before routine clinical implementation.

## References

[1] Harrison CN, Butt N, Campbell P (2013). Diagnostic pathway for the investigation of thrombocytosis. Br J Haematol.

[2] Arber DA, Orazi A, Hasserjian R (2016). The 2016 revision to the World Health Organization classification of myeloid neoplasms and acute leukemia. Blood.

[3] Bleeker JS, Hogan WJ (2011). Thrombocytosis: diagnostic evaluation, thrombotic risk stratification, and risk-based management strategies. Thrombosis.

[4] Tian Y, Zong Y, Pang Y, Zheng Z, Ma Y, Zhang C, Gao J (2025). Platelets and diseases: signal transduction and advances in targeted therapy. Signal Transduct Target Ther.

[5] Tefferi A, Barbui T (2015). Essential thrombocythemia and polycythemia vera: focus on clinical practice. Mayo Clin Proc.

[6] Bader MS, Meyer SC (2022). JAK2 in myeloproliferative neoplasms: still a protagonist. Pharmaceuticals (Basel).

[7] Müskens JL, Kool RB, van Dulmen SA, Westert GP (2022). Overuse of diagnostic testing in healthcare: a systematic review. BMJ Qual Saf.

[8] Noris P, Melazzini F, Balduini CL (2016). New roles for mean platelet volume measurement in the clinical practice?. Platelets.

[9] Budak YU, Polat M, Huysal K (2016). The use of platelet indices, plateletcrit, mean platelet volume and platelet distribution width in emergency non-traumatic abdominal surgery: a systematic review. Biochem Med (Zagreb).

[10] Hafeez A, Nasir ud Din Khattak, Naeem S, Robert HM, Mohsin S, Mahmood A (2023). Platelet indices as a tool for differentiation between clonal thrombocytosis and reactive thrombocytosis. Pak Armed Forces Med J.

[11] Selvaraju V, Jayaganesh Jayaganesh, Nikshayaa Nikshayaa (2023). Platelet indices in differentiating reactive versus clonal thrombocytosis. Saudi J Pathol Microbiol.

[12] Shen CL, Hsieh TC, Wang TF, Huang WH, Chu SC, Wu YF (2021). Designing a scoring system for differential diagnosis from reactive thrombocytosis and essential thrombocytosis. Front Med (Lausanne).

[13] Al-Tameemi WF, Noori AK (2022). The impact of platelet indices in the evaluation of different causes of platelet count disorder. Iraqi J Hematol.

[14] Sillen M, Declerck PJ (2021). Thrombin activatable fibrinolysis inhibitor (TAFI): an updated narrative review. Int J Mol Sci.

[15] Muhammed Safwan KS, Prajna KM, Ullas C (2024). A clinicopathological evaluation on thrombocytosis in correlation with platelet indices in a tertiary care hospital: a retrospective study. Int J Res Med Sci.

